# Attitudes and stigma toward seeking psychological help among Saudi Adults

**DOI:** 10.1186/s40359-022-00923-4

**Published:** 2022-09-15

**Authors:** Bushra A. Alluhaibi, Abdel W. Awadalla

**Affiliations:** 1 Maternity and Children’s Hospital, The Holy Capital of Makkah, Ministry of Health, Makkah, Kingdom of Saudi Arabia; 2 Department of Psychiatry, College of Medicine, King Fahad University Hospital, Imam Abdulrahman Bin Faisal University, Dammam, 31952 Kingdom of Saudi Arabia

**Keywords:** Attitudes, Distress, Mental illness, Seeking psychological help, Stigma, Saudi Arabia

## Abstract

**Background:**

In the Kingdom of Saudi Arabia (KSA), public attitudes and stigma toward mental health illness seem to prevent people from seeking psychological help, which negatively impacts an individual's life. The primary objective of this study was to investigate people's attitudes toward seeking psychological help and identify the extent to which the associated stigma is responsible for preventing them from seeking psychological help.

**Methods:**

Two hundred eighteen adults recruited from the community living in the Eastern Province of the KSA completed the questionnaires, customized to create the Arabic version of Attitudes Toward Seeking Professional Psychological Help Scale-Short Form (ATSPPH-SF-A), the Arabic version of Stigma Scale for Receiving Psychological Help (SSRPH-A), and the Arabic version of Hopkins Symptom Checklist-25 (HSCL-25-A).

**Results:**

Both stigma and psychological distress significantly affected attitudes toward seeking professional help. Furthermore, it indicated that attitudes were negatively correlated with stigma while positively correlated with psychological distress. No significant difference in attitudes toward psychological help-seeking was identified between male and female participants. However, males displayed higher levels of stigma, while females showed greater psychological distress. Furthermore, the groups who received psychological treatment demonstrated more favorable attitudes toward seeking psychological help.

**Conclusion:**

Stigma and psychological stress influence attitudes toward treatment-seeking behavior for mental illness, making them two major predictors responsible for the underutilization of mental health services. More research is needed to assess specific sociodemographic disparities across more data sources and the factors that further contribute to stigma and psychological distress.

## Background

The World Health Organization has reported that approximately 25% of the world's population suffers from some form of mental illness, with depression and anxiety being the most common conditions [1]. At primary healthcare centers (PHCs), the prevalence rate of psychiatric disorders is estimated to be 30–46%, with depression accounting for 20% of the total in the KSA [2]. Saudi women and elderly individuals with any form of disability recorded a higher prevalence rate [3–5].

Mental illness (henceforth, MI) can cause severe distress and disability that impair the quality of life of the individual [6]. Although a treatable condition if diagnosed at an early stage, the stigma and negative attitudes toward MI inhibit patients from seeking psychological help and impede their recovery from mental disorders [7].

### Mental health stigma

Mental health stigma is defined as the disgrace, societal disapproval, or social discrediting of individuals who live with mental health problems [8]. It often involves society's general fear, lack of respect and refusal to accept a person living with MI [9].

Multiple dimensions and theoretical models of stigma coexist. One of the early definitions of stigma was explored by Durkheim [10] as a social phenomenon and more recently as a negative social identity that often results in a person being disqualified from full social acceptance [11–13]. Subsequent work [14, 15] categorized stigma into two distinct types of public stigma and self-stigma that may work together to form a barrier to seeking mental health services.

### Psychological help-seeking stigma

From a help-seeking perspective, the perception of the multifaceted nature of stigma may lead individuals to avoid psychological help-seeking [16]. For example, public stigma is defined as the prevalence of negative beliefs held by the general public toward persons with MI as undesirable or socially unacceptable [11]. Corrigan and Watson [17] have classified it as a composite of three distinct components: stereotypes, prejudice and discrimination; together, they tend to prevent persons with MI and their families from utilizing psychological help-seeking. On the other hand, self-sigma occurs when an individual internalizes these negative beliefs against themselves, which, in turn, evoke negative emotional and behavioral reactions [18]. Similar to self-stigma, label avoidance is frequently framed as a consequence of public stigma, which occurs when individuals refrain from seeking psychological help to avoid negative labels [19]. In other words, stigmatized persons might feel responsible for their own MI, and therefore, may bear greater feelings of shame and hesitation toward seeking psychological help [20]. Researchers have noted that attitudes toward seeking help are a powerful indicator of the intention to seek psychological help, as they affect the individual's decision to seek help when dealing with a psychological problem [21–23].

As part of the Kingdom’s Vision 2030, the Saudi health authority intends to provide more counseling services to facilitate the primary mental healthcare system by increasing mental health clinics and clinicians across the Kingdom. It also attempts to promote mental health literacy (MHL) by providing knowledge about the causes, recognition and management of MI that lead to more effective treatment [24, 25]. One study that supports this approach was targeted at college students by Cheng and collaborators [26]. The authors found that MHL was able to help students identify symptoms of depression and anxiety and develop positive attitudes toward seeking psychological help. Hence, familiarity with MI tends to be associated with more positive attitudes and a willingness to seek psychological help [27].

However, despite significant strides to improve knowledge and awareness of help-seeking behavior, underlying negative attitudes among the Saudi general public toward persons with MI still exist. A significant proportion of the population still chooses not to seek psychological help when suffering from psychological distress. In a recent study conducted among the general Saudi population [28], knowledge about MI was found to be poor and associated with increased negative perceptions and attitudes toward seeking psychological help*.* Another similar study found that nearly half of college participants tended to hold negative attitudes toward mentally ill persons and the management of MI [29]. In a large-scale study undertaken by Almubark and colleagues [30], approximately half (46%; n = 3557) of surveyed participants reported little knowledge about MHL. The authors attributed risk factors to persons who were elderly, had low income or lack of education, were formerly married and demonstrated a moderate pattern of utilization of health services. Akin to other Gulf Arab states [31], Saudi persons with disabilities may represent a stigma for their families. Women are particularly affected by being ignored or hidden from taking part in social events out of fear of having a negative impact on the marriage prospects of siblings or relatives [32, 33]. This may represent a more realistic view of a family member’s affiliate stigma [34] identified across various sociocultural contexts [9, 19, 35–37]. The patient’s families often internalize the negative consequences (e.g., shame, embarrassment, anger) linked to discriminatory public attitudes toward persons with MI [20].

In a review of the extant literature, it was observed that few attempts have investigated attitudes, stigma and knowledge toward seeking professional help in the KSA [7, 28]. These attempts identified attitudinal stigma and poor knowledge of MI as commonly reported barriers to mental health services among the Saudi public. None of these studies specifically investigated the stigma of psychological help-seeking and its patterns in sociocultural contexts. Therefore, the purpose of the present study was to investigate Saudi adults' attitudes toward seeking psychological help and assess the level of stigma and other associated symptoms. Our study was guided by the research question that stigma, attitudes and stress play a significant role in hindering the process of seeking psychological help. Based on the findings of previous studies [2, 7], we hypothesized that (1) participants who received previous psychological treatment would display more positive attitudes toward psychological help-seeking, (2) participants’ attitudes toward psychological help-seeking would negatively relate to the stigma of help-seeking and positively to psychological stressors (3) women would report higher perceived stigma than men as measured by SSRPH-Arabic, and (4) psychological distress would positively relate to help-seeking attitudes.

## Method

### Participants and setting

This cross-sectional study was carried out among 221 Saudi adults living in the Eastern Province of the KSA. A post hoc power analysis was conducted using G*Power version 3.1.9.6 [38] to determine the minimum sample size required to test the study hypotheses. Our analysis indicated that the required sample size to achieve 80% power for detecting a medium effect, at a significance criterion of α = 0.05, was N = 219 (r = 0.19). Thus, the obtained sample size of N = 221 is adequate to test the study hypotheses.

The participants eligible were recruited by convenient sampling from public places (coffee shops, universities, malls, supermarkets, hospitals, mosques, etc.). They were informed of the purpose of the study and voluntary participation, including no penalty if they decided not to participate or withdraw from the study at any time without giving a reason. Three (1.4%).

participants did not meet the inclusion criteria of reading ability and were therefore excluded from the study. Hence, the data were collected from 218 participants who fulfilled the study's eligibility criteria (18 ≥ years old, of both genders, literate in the Arabic language, and willing to participate). The study was conducted as per the norms of the Declaration of Helsinki. Before starting the study, ethical approval (IRB –PGS –2021 –01–449) was obtained from the Ethics Committee of Imam Abdulrahman Bin Faisal University in Dammam. Data were collected after obtaining written consent from the participants.

#### Data collection and measures

Data were collected by using a data collection sheet consisting of four sections. The first section contained sociodemographic information, that is, gender, age, educational level, occupation, marital status, and socioeconomic status (SES). Participants were also classified based on whether they had previously received help from a mental health provider.

#### Attitudes toward seeking professional psychological help scale-short form (ATSPPH-SF-Arabic)

The second section consisted of the Arabic version of the Attitudes Toward Seeking Professional Psychological Help Scale-Short Form (ATSPPH-SF-A) [31], which is a 10-item scale evaluating the attitudes of participants toward seeking professional help for psychological problems [39]. The items are rated on a 4-point Likert-type scale ranging from 0 (“disagree”) to 3 (“agree”). For example, “I would want to get help if I were worried or upset for a long period of time”. The items are then summed up, with higher scores indicating more positive attitudes toward psychological help. The total ATSPPH-SF-A score is obtained by reversing half of items 2, 4, 8, 9 and 10. The instrument was validated in a sample of Emirati (UAE) college students, with a reported low Cronbach’s alpha of 0.65 and then 0.67 in a subsequent study by Vogel and colleagues [23]. The current study obtained a similar low Cronbach’s alpha value of 0.65.

#### Stigma scale for receiving psychological help (SSRPH-Arabic)

The third section comprised the Arabic version of the Stigma Scale for Receiving Psychological Help (SSRPH-A) [31, 40]. It is a 5-item self-reported instrument that measures the stigma associated with receiving psychological help. Items are rated on a 4-point Likert-type scale ranging from 1 ("strongly disagree") to 4 ("strongly agree") [40]. For example, the items included “It is advisable for a person to hide from people who he/she has seen a psychologist” and “people tend to like less those who are receiving professional psychological help”. The SSRPH-A total score is obtained by summing up the five items, with higher scores indicating a higher degree of perceived stigma. The instrument was validated in a sample of Emirati college students, with an acceptable reported alpha of 0.70 [31]. The current study obtained a similar satisfactory Cronbach’s alpha value of 0.72

#### Hopkins symptom checklist-25 (HSCL-25-Arabic

The last section was the Arabic version of the Hopkins Symptom Checklist-25 (HSCL-25—A) validated in a sample of Palestinian college students to measure the presence and intensity of anxiety and depression symptoms (e.g., fear, self-blame) [41]. The HSCL-A is a 25-item self–report questionnaire that consists of two parts: part one has 10 items for anxiety symptoms, and part two has 15 items for depression symptoms. The scale for each question includes four response options (i.e., "Not at all", "A little", "Quite a bit", "Extremely," rated 1 to 4, respectively). The rating is based on the reported symptoms over the past week. Responses are summed and divided by the number of answered items to generate scores for anxiety and depression symptoms. Individuals with scores on anxiety and/or depression greater than 1.75 are considered symptomatic, whereas 1.75 is the cutoff point for scientific validity. It was demonstrated that the overall scores of the inventory are related to the acute emotional distress of an undetermined diagnosis [42]. The Arabic validation sample excluded item 14, "Loss of sexual interest or pleasure", as this was culturally inappropriate to be examined. Thus, the scale was 24 items, and the obtained Cronbach's alpha value was 0.90 [341]. In our study, we added the excluded item and obtained a high alpha value of 0.92***.***

#### Data analysis

Statistical analysis was performed using the Statistical Package of Social Sciences (SPSS) version 22.0. Frequencies and percentages were calculated to describe the data. One-way ANOVA was used to compare the stigma, psychological distress, and attitudes toward psychological help-seeking for independent variables containing multiple categories such as age groups, occupation, and marital status. An independent t-test was used for statistical testing of variables with only two categories (e.g., gender and past history of seeking treatment). Pearson's correlation was calculated to find a correlation between stigma, psychological distress, attitudes toward psychological help-seeking and symptoms of anxiety and depression. All the factors that were statistically significant at the univariate level were included in the multiple linear regression that influenced the attitude of a person toward seeking psychological help. Reliability statistics were concluded using Cronbach's alpha value. The tests of post hoc hypotheses were assessed using a one-way ANOVA with a Bonferroni adjusted alpha level of 0.0125 per test (0.05/4). The results of the pairwise comparison indicated an appropriate *P* value level between age group and HSL (α = 0.0125), occupation and HSL (α = 0.007), and marital status and SSRPH (α = 0.017).

## Results

Table 1 shows that out of 218 participants, 100 (45.9%) were males, and 118 (54.1%) were females. Their ages ranged from 18 to 77 years, with a mean age of 33.9 (SD ± 11.9) years. The majority of the participants (n = 167, 76.6%) held a college degree and above. Approximately 58 (26.6%) were professionals, 54 (24.8%) were technicians, 35 (16.1%) were students, 28 (12.8%) were housewives, 21 (9.6%) were unemployed, 15 (6.9%) were retired, and 7 (3.2%) ran private businesses. More than half of the participants were married (n = 119, 54.6%) young adults (26–40 years; 53.7%) with middle-income socioeconomic status (n = 193, 88.5%). Only 23 (10.6%) of the participants had received treatment in the past.

**Table 1:** Sociodemographic and clinical characteristics of participants (n = 218)

Variables	(N = 218)	
	n	n%
*Gender*		
Male	100	45.9
Female	118	54.1
*Age groups*		
18-25yrs	54	24.8
26-40yrs	117	53.7
41-59yrs	33	15.1
60 and older	14	6.4
*Education*		
Intermediate	8	3.7
Diploma	43	19.7
College	141	64.7
Graduate	26	11.9
*Occupation*		
Student	35	16.1
Technician	54	24.8
Professional	58	26.6
Private Business	7	3.2
Unemployed	21	9.6
Retired	15	6.9
Housewife	28	12.8
*Marital status*		
Single	86	39.4
Married	119	54.6
Divorced/Widowed	13	5.9
*SES*		
Low	14	6.4
Middle	193	88.5
High	11	5.0
*Previous treatment*		
Non-received	195	89.4
Received	23	10.6

As described in Table 2, the participants who received previous psychological treatment showed a significantly and positively higher attitude toward seeking psychological help and significantly higher psychological distress than those who did not receive treatment. The amount of stigma for seeking psychological help was significantly higher among males than the female participants (*p* = 0.006). However, it was lower among divorced/widowed participants than among single or married participants. The multiple comparison analysis with *p* < 0.017 being significant showed that divorced/widowed was significantly different from married. Psychological distress was higher among females than males. It was also higher in the age group 18–25 years compared to the other age categories, while the multiple comparison analysis with *p* < 0.0125 being significant showed that 18–25 years was significantly different from 26 to 40 years. The highest level of psychological distress was found among the participants who were in private business compared to the rest of the categories.

**Table 2:** Mean comparison of attitudes toward seeking psychological help, stigma, and psychological distress among sociodemographic and clinical characteristics of participants

Variables	ATSPPH-SF		SSRPH		HSL-25	
	Mean ± SD	*P* value	Mean ± SD	*P* value	Mean ± SD	*P* value
*Gender*		0.559		0.006*		0.005*
Male	1.57 ± 0.54		2.15 ± 0.60		1.66 ± 0.45	
Female	1.61 ± 0.50		1.93 ± 0.54		1.85 ± 0.46	
*Age groups*		0.265		0.101		0.004*
18-25yrs	1.51 ± 0.52		1.99 ± 0.51		1.94 ± 0.49	
26-40yrs	1.60 ± 0.53		2.06 ± 0.60		1.68 ± 0.41	
41-59yrs	1.57 ± 0.54		2.15 ± 0.60		1.82 ± 0.50	
60 and older	1.82 ± 0.43		1.71 ± 0.52		1.56 ± 0.42	
*Education*		0.555		0.950		0.244
Intermediate	1.76 ± 0.24		2.13 ± 0.50		2.03 ± 0.46	
Diploma	1.54 ± 0.50		2.00 ± 0.56		1.80 ± 0.47	
College	1.58 ± 0.53		2.04 ± 0.57		1.75 ± 0.45	
Graduate	1.68 ± 0.55		2.04 ± 0.67		1.67 ± 0.50	
*Occupation*		0.283		0.576		0.043*
Student	1.41 ± 0.56		1.98 ± 0.44		1.91 ± 0.42	
Technician	1.57 ± 0.45		2.11 ± 0.63		1.80 ± 0.52	
Professional	1.61 ± 0.56		2.00 ± 0.56		1.68 ± 0.36	
Private Business	1.73 ± 0.84		2.37 ± 0.58		1.98 ± 0.58	
Unemployed	1.67 ± 0.51		2.00 ± 0.60		1.68 ± 0.44	
Retired	1.79 ± 0.46		1.89 ± 0.54		1.49 ± 0.35	
Housewife	1.59 ± 0.42		2.02 ± 0.66		1.83 ± 0.52	
*Marital status*		0.369		0.016*		0.096
Single	1.53 ± 0.53		2.03 ± 0.50		1.84 ± 0.46	
Married	1.63 ± 0.52		2.08 ± 0.62		1.70 ± 0.46	
Divorced/Widowed	1.63 ± 0.48		1.60 ± 0.51		1.79 ± 0.43	
**SES**		0.543		0.056		0.080
Low	1.56 ± 0.45		1.97 ± 0.59		1.68 ± 0.51	
Middle	1.60 ± 0.51		2.02 ± 0.57		1.75 ± 0.45	
High	1.43 ± 0.78		2.44 ± 0.63		2.07 ± 0.51	
*Previous treatment*		0.014*		0.831		0.004*
Non-received	1.56 ± 0.51		2.04 ± 0.57		1.72 ± 0.42	
Received	1.86 ± 0.57		2.01 ± 0.65		2.20 ± 0.64	

*Indicates a significant *p*-value of less than 0.05

Table 3 shows the Pearson correlation coefficient, the correlation between stigma, psychological distress, and attitudes toward psychological help-seeking. Consistent with expectations, this revealed that attitudes toward seeking psychological help were significantly negatively correlated with stigma (r = –0.242, *p* < 0.01) and significantly positively correlated with psychological distress (r = 0.186, *p* < 0.01). Briefly, contrary to expectations, stigma was significantly positively correlated with psychological distress (r = 0.197, *p* < 0.01), and with depression symptoms (r = 0.229, *p* < 0.01).

**Table 3:** Means, standard deviations, alpha coefficients, and correlations for all variables

Variables	Help-seeking (ATSPPH-SF)	Stigma (SSRPH-A)	Psychological distress (HSL-25)	Anxiety symptoms	Depression symptoms
Help-seeking (ATSPPH-SF)	1	− 0.242*	0.186*	0.184*	0.162*
Stigma (SSRPH-A)		1	0.197*	0.077	0.229*
Psychological distress (HSL-25)			1	–	–
Anxiety symptoms				1	–
Depression SYMPTOMS					1
Mean	1.57	2.15	1.66	1.83	1.74
SD	0.54	0.60	0.45	0.52	0.50

*Indicates significant *p*-value of less than 0.05

Table 4 represents the multiple linear regression, where the calculation was used to test whether stigma, psychological distress and previous psychological treatment significantly predicted participants' attitudes toward seeking psychological help. The results of the regression indicated that the predictors explained 12.3% of the variance [adjusted R^2^ = 0.123 (F (3,194) = 10.210, *p* = 0.001]. It was found that stigma (β =  − 0.28, *p* = 0.001), psychological distress (β = 0.20, *p* = 0.005) and previous treatment (β = 0.15, *p* = 0.031) significantly predicted attitudes.

**Table 4:** Multiple linear regression model showing the factors that are associated with attitude toward seeking psychological help

Variable	B	95% CI	Beta	t	*P* value	*R*^*2*^	Adj*R*^*2*^
Stigma	− 0.243	− 0.361, − 0.124	− 0.275	− 0.4027	0.001	0.136	0.123
Psychological distress	0.227	0.068, 0.386	0.199	2.808	0.005		
Previous treatment	0.279	0.026, 0.532	0.151	2.172	0.031		

## Discussion

The purpose of the present study was to examine the attitudes of people toward seeking psychological help, evaluate the stigma that prevents them from seeking such help, and the relationship between psychological distress, attitudes and stigma toward seeking psychological help.

### Help-seeking Attitudes

The literature suggests that attitudes toward seeking psychological help are a strong indicator of behavior and intention to seek psychological help when confronted with an individual mental problem [43, 44]. Our study found that the participants who previously received psychological treatment displayed more positive attitudes toward seeking psychological help than those who did not receive treatment [45], which confirms our hypothesis. However, the proportion of those who sought psychological help was markedly small (10.6%), perhaps suggesting participants’ insufficient information on MI [30], distress level [46], or physicians’ poor knowledge about MHL. Al-Atram [47] emphasized the persistence of negative attitudes toward people with MI among Saudi mental health professionals. The author investigated knowledge and attitudes toward MI among 142 healthcare practitioners from various health fields, and the results showed that nearly two-thirds (63.9%) of participants had never referred any case to a mental health provider*.* Except for only a small proportion of family practitioners (8.3%), the majority (86.3%) of practitioners reported having no interest in a mental health provider. Such findings point to a pressing need for further examining the beliefs about MI and MHL within the sociocultural context of Saudi laypersons. Thus, this can be achieved if mental health providers and policymakers can address two major barriers to psychological help-seeking utilization: the stigma and general attitudes of individuals and society as a whole. In addition, efforts are required to increase knowledge about MI among Saudi healthcare professionals, as education has consistently been linked to more favorable attitudes and readiness to make social contacts with persons with MI [20, 27]. Surprisingly, in our study, education and socioeconomic variables failed to predict negative attitudes toward psychological help-seeking. This was consistent with previous research findings indicating that the two variables could predictably affect other social processes but had an inconsistent and rarely significant effect on stigma or negative attitudes [48, 49].

In the literature review of previous studies, no significant gender differences were found in terms of attitudes toward seeking psychological help in one study [50, 51]. In contrast, other researchers found a significant difference between attitude and gender, as women tended to have more favorable attitudes and a willingness to seek psychological help [15, 52, 53]. This was reversed in Khan et al.'s [50] study, where women with internalizing stigma tended to consistently experience discrimination and social withdrawal that likely evoked negative attitudes toward seeking psychological help. Such discrepant findings may be linked to the use of different study designs or research measures that render incomparable results.

### Stigma and help-seeking attitudes

Our findings showed that participants’ attitudes toward psychological help-seeking were significantly and negatively related to the stigma of help-seeking and positively related to psychological stressors, which confirms our hypothesis. This was consistent with what has been found in previous studies [21, 23, 44, 52].

Gender differences were identified at the level of stigma perceived for seeking help, showing that males had a higher level of perceived stigma than females, which did not support the hypothesis. This result could be explained within the setting of the Saudi sociocultural context, whereby if a man has a psychological problem, asking for help outside the family may be seen as a sign of weakness or a source of shame to himself or his family [54]. One of the reasons why men do not ask for psychological help is that it does not fit with their cultural beliefs about masculinity and acceptable male behavior [55]. Chatmon [56] referred to this as toxic masculinity, which may lead to difficulty in expressing emotions and restriction in behaviors. Furthermore, the relationship between the stigma of psychological help-seeking and the recognition of the need to seek help may reflect cultural beliefs that psychological help-seeking is shameful, and that issues must be solved by oneself or within the family [19, 41]. Abdullah and Brown [54] suggested that those who seek mental health services are often encouraged to reveal their emotions. However, the self-disclosure required for help-seeking may violate the value of family honor and yield more negative consequences connected with self-stigma and discrimination in social relationships, employment and healthcare [20]. The results may be explained by the role of traditional male gender expectations, which discourage expressions of emotion and pain, and demand strength and self-reliance [19, 39].

Interestingly, the study found that stigma toward psychological help-seeking was higher among married persons. This could be explained by the fear of violating the honor of the family and the concern that seeking psychological help would indicate personal weakness and bring shame to marital relations. Studies in the Saudi environment have found that most persons with MI and their relatives consider these perceptions to be an obstacle for married people who require psychological help [24]. Our study showed that a smaller percentage (10.6%) of the total sample had received previous treatment. Hence, one might assume that fewer married participants had received psychological help in the past.

Stigma was significantly and positively correlated with psychological distress, specifically with depressive symptoms. Whenever the perception of stigma increased, psychological distress also increased, as indicated in previous studies reviewed to understand mental health illnesses [40]. Moreover, Saudi society embodies an Islamic culture that associates a person’s suffering or illness with the Will of God. It is believed that only regular prayers, healing rituals (e.g., incantation, cauterization, cupping), or a sense of personal responsibility can help treat such conditions [57]. Some scholars [20, 58] argued that the explanations attributed to the internalizing factor of self-responsibility could lower the general public's willingness to interact with persons with MI. It is suggested that this may focus the problem more on an individual's cognitive processing of information rather than on his or her subjective experience of stigmatization such as discrimination and isolation. However, this result needs further study to identify the differences between self-stigma and social stigma, improve education about and awareness of MI in the community and health institutions, treat it as a physical illness, and overcome the fear of people suffering from mental health problems.

### Psychological distress and help-seeking attitudes

Expectedly, psychological distress was a significant predictor of psychological help-seeking attitudes. However, this relationship was found to be positive, as increased distress was associated with more positive help-seeking attitudes within the sample. Specifically, the results supported the hypothesis of the study [47, 53]. One study found that psychological distress was a positive predictor of attitudes toward seeking psychological help [41].

Wide gender differences were detected at the level of distress, which found that women display higher levels of psychological distress and a tendency to seek psychological help. In contrast, men are less likely to seek psychological help and therefore employ adaptive coping mechanisms (e.g., exercising, keeping busy) to relieve emotional and physical pain [59]. These gender differences could be explained by two categories of coping behavior, as women are more emotionally focused on appraising threatening events, whereas men use more problem-focused methods of altering or eliminating stressful experiences [43, 60].

Furthermore, sociocultural pressures are often more influential over women [61]. For example, in a male-dominated society, women are expected to comply with two sets of values: domestic and professional. This results in a conflict of roles, which likely leads to more emotional distress and a tendency to seek psychological help. Denton and associates [46] found exposure to stress in social life, financial stress, parenting stress, environmental stress, family health stress, and job stress to be positively associated with psychological distress. Our analysis showed that being a married woman aged 26–40 years, being employed, and having a previous history of psychological help-seeking was associated with a considerably high level of distress. Surprisingly, education and SES levels insignificantly emerged as contributing factors to stressful conditions among these women. Aldossari [5] provided several potential reasons that place Saudi women at a higher risk of psychological distress and poor health. These reasons include family caregiving roles, mobility restrictions, economic hardship, and marital conflicts, among others.

However, new legislation for improving women's rights was implemented by the Saudi government. It is based on granting permission to drive, providing more work opportunities and loosening restrictions on mobility and travel abroad without approval from a *mahram* (husband or male relative whom she is prohibited from marrying) [62]. These reforms markedly increased the presence of female participation in the workforce as the rate climbed from 18.6% in 2011 to 23.37% in 2018 [63]. During the first half of 2021, 59% of Saudi citizens recruited within the private sector were women [64].

Interestingly, our study found that those in the 18- to 25-year-old age group were mostly students and had high levels of psychological distress. This is likely because the sample was collected during the time of student examinations. Based on this finding, we predict that the severity of psychological distress may positively influence these students to seek psychological help [44]. This finding has also been reported and supported by other studies [65]. Finally, the study found that people who had private businesses had the highest rate of psychological distress caused by the extra workload associated with running a business. This was consistent with a similar study conducted in South Africa [66, 67].

## Limitations and Strengths

The main limitations of the study were its cross-sectional nature and the fact that the collected data were specific to one region, which may restrict the generalizability of the results. In addition, the study sample was relatively small. Hence, further analysis with a larger sample size would be useful to determine the replicability of these findings with Saudi adults. Furthermore, the study was based solely on self-report instruments, which may have a limitation due to potential participant bias in the indirect reporting of their behavior, beliefs, attitudes or intentions, as they were more likely to report experiences that were considered culturally and socially acceptable or preferred to their conservative community. In addition, the low level of an alpha coefficient (α = 0.65) for ATSPPH-SF-A could possibly be explained by participants’ increased false positives due to reverse-coded items requiring more time to be processed [67]. Moreover, the researchers used a nonvalidated, single-item question on the clinical questionnaire to identify people who had previously received treatment, although there should be other clinical questions to identify what they were suffering from to determine their correlation with the variables.

Notwithstanding all the limitations, our study has several clinical and scientific implications, including a high response rate to the study questionnaire (98.6%). The main contribution of this study to the literature on psychological help-seeking attitudes was the finding of a significant interaction of stigma and psychological distress, as well as distress as defined by the expression of symptoms (HSCL-25-A), in explaining the predictor variables in help-seeking attitudes. The focus of this study was attitudes toward seeking psychological help, as insufficient research has been conducted with respect to Saudi adults. In contrast to previous studies, this study selected participants from the general public, the instruments used in this study were in Arabic, and the alpha coefficient was acceptable. This study found that, at high levels of distress, individuals tended to have significantly positive attitudes toward seeking psychological help. However, high levels of stigma significantly altered help-seeking attitudes to negative. This is an important conclusion given that most people tend to only seek help when experiencing considerable psychological distress. More research is needed to assess specific sociodemographic disparities across more data sources. Further analyses are also needed to assess the influence of these mental health challenges on the daily lives of the people they impact.

## Conclusion

The current study reported the presence of a statistically significant inverse relationship between attitudes and stigma of help-seeking and a positive relationship with psychological distress. The results of this study could contribute to the literature that addresses the underutilization of help-seeking services by stigmatized persons with MI and their families. Certainly, there is a need for an increased understanding of Saudi sociocultural aspects of health stigma and its impact on help-seeking attitudes.

## Data Availability

The datasets generated and/or analyzed in the present study are available from the corresponding author on reasonable request.

## Abbreviations

ANOVA: Analysis of variance
ATSPPH-SF-A: Attitudes toward seeking professional psychological help scale-short form
HSCL-25-A: Hopkins symptom checklist-25
KSA: Kingdom of Saudi Arabia
MHL: Mental health literacy
MI: Mental illness
PMC: Primary healthcare
SES: Socioeconomic status
SSRPH-A: Stigma scale for receiving psychological help
UAE: United Arab Emirates
